# New Steroids from the Soft Coral *Nephthea chabrolii*

**DOI:** 10.3390/md11020571

**Published:** 2013-02-22

**Authors:** Shang-Kwei Wang, Shyh-Yueh Puu, Chang-Yih Duh

**Affiliations:** 1 Asia-Pacific Ocean Research Center, National Sun Yat-sen University, Kaohsiung 804, Taiwan; E-Mail: skwang@cc.kmu.edu.tw; 2 Department of Microbiology, Kaohsiung Medical University, Kaohsiung 807, Taiwan; 3 Department of Marine Biotechnology and Resources, National Sun Yat-sen University, Kaohsiung 804, Taiwan; E-Mail: a9401098@stmail.isu.edu.tw

**Keywords:** *Nephthea chabrolii*, 19-oxygenated steroid, norergosterol, cytotoxicity

## Abstract

A new cytotoxic 19-oxygenated steroid, nebrosteroid Q (**1**) and two new cytotoxic 19-norergosterols, nebrosteroids R and S (**2** and **3**) were isolated from the soft coral *Nephthea chabrolii* collected at San-Hsian-Tai. The structures of nebrosteroids Q–S (**1**–**3**) were elucidated by spectral analysis, and their cytotoxicity against selected cancer cells as well as antiviral activity against human cytomegalovirus (HCMV) were measured *in vitro*.

## 1. Introduction

**Figure 1:**
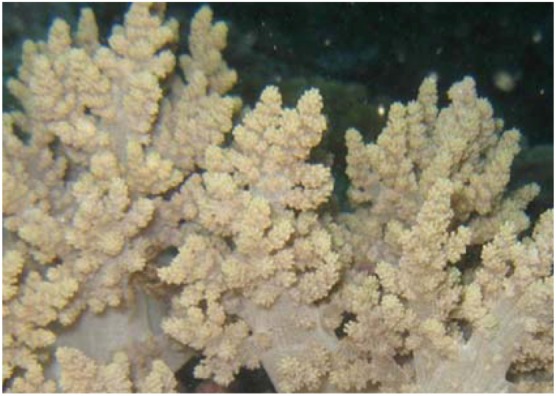
Soft coral *Nephthea chabrolii*.

**Figure 2:**
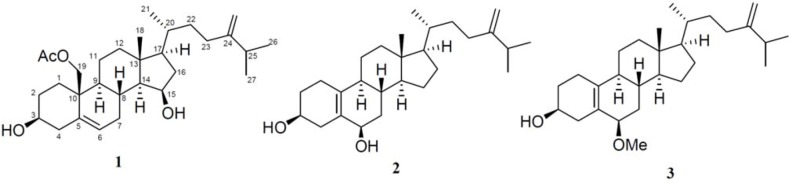
Structures of compounds **1**–**3**.

Numerous secondary metabolites including steroids, sesquiterpenoids, diterpenoids, and meroditerpenoids have been isolated from soft corals of the genus *Nephthea* [1,2,3,4,5,6,7,8,9,10,11,12,13,14,15,16,17,18,19,20,21,22,23,24,25,26]. Previous studies on these materials showed them to exhibit diverse biological properties including cytotoxic [3,4,5,6,17,19,26], anti-inflammatory [12,13,22,25] and antimicrobial activities [18]. The acetone extract of the soft coral *Nephthea chabrolii* (Figure 1) was found to be cytotoxic towards P-388 mouse lymphocytic leukemia cells. Chromatographic fractionation led to the isolation of a new cytotoxic 19-oxygenated steroid, nebrosteroid Q (**1**) and two new cytotoxic 19-norergosterols, nebrosteroids R and S (**2** and **3**) (Figure 2).

## 2. Results and Discussion

**Figure 3:**
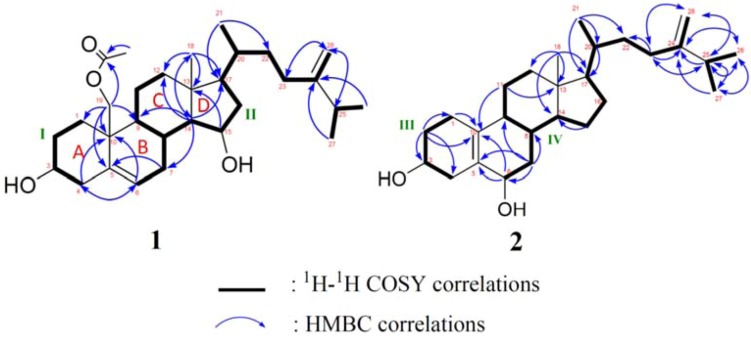
COSY and HMBC correlations of compounds **1** and **2**.

**Figure 4:**
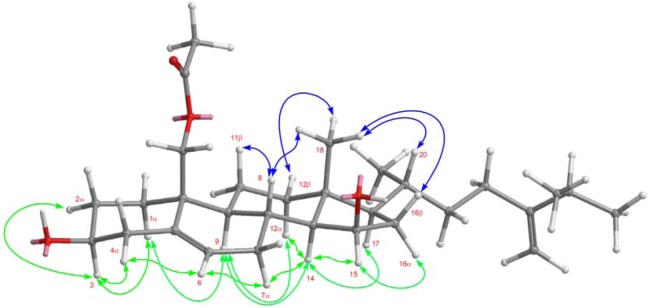
NOESY correlations of compound **1**.

Nebrosteroid Q (**1**) had a molecular formula of C_30_H_48_O_4_ as established by interpretation of its HRESIMS and NMR data (Table 1). The IR spectrum of **1** indicated the presence of hydroxyl(s) (ν_max_ 3375 cm^−1^) and ester groups (ν_max_ 1737 cm^−1^). Further, the ^1^H NMR data (in CD_3_OD) revealed the presence of a tertiary methyl (δ_H_ 0.97), three secondary methyls (δ_H_ 0.97, 1.02, and 1.03), two oxymethines [δ_H_ 3.44 (1H, m), 4.14 (1H, td, *J* = 5.6, 2.0 Hz)], and an oxymethylene [δ_H_ 4.02, 4.53 (*J*_AB_ = 12.0 Hz)]. The presence of a trisubstituted double bond was revealed by NMR data (in CD_3_OD) [δ_H_ 5.62 (1H, d, *J* = 5.6 Hz), δ_C_ 126.7 (CH), 137.2 (C_q_)] (Table 1). NMR data (in CD_3_OD) of **1** exhibited the presence of an acetoxyl group [δ_H_ 2.04 (3H, s), δ_C_ 21.1 (CH_3_), 172.7 (C_q_)]. The ^13^C NMR and DEPT spectra of **1** contained resonances for ten sp^3^ methylenes, eight sp^3^ methines, two quaternary sp^3^ carbons, one sp^2^ methine, one sp^2^ methylene, two quaternary sp^2^ carbons, and one carbonyl. Comparison of NMR chemical shift values of **1** with those of ergost-5-en-3β,15β,19-triol [5] reported from the soft coral *Nephthea erecta* as well as its HMBC cross-peaks of H_2_-19/C-1, C-5, C-9, C-10, carbonyl carbon at C-19 suggested that **1** may be a 19-acetyl analogue of ergost-5-en-3β,15β,19-triol. Interpretation of the ^1^H-^1^H COSY spectrum led to partial structures I and II (Figure 3). Rings A and B were elucidated on the basis of HMBC cross-peaks (Figure 3) between H_2_-19/C-1, C-5, C-9, C-10 and H_2_-4, H-6/C-10, whereas rings C and D were completed based on HMBC correlations between H_3_-18/C-12, C-13, C-14, C-17. The NOESY correlations (in CDCl_3_) (Figure 4) observed between H-11β and H_3_-18, H-11β and H-19, H-19 and H-4β, H_3_-18 and H-8, H_3_-18 and H-20, H-3 and H-4α, H-9 and H-14, H-15 and H-16α, and H-15 and H-14 in **1** confirmed that nebrosteroid Q (**1**) was ergost-5-en-3β,15β,19-triol 19-acetate.

**Table 1:** ^1^H and ^13^C NMR data for compounds **1**–**3**.

position		1		2		3	
	δ_H_^a^ (*J* in Hz)	δ_H_^b^ (*J* in Hz)	δ_C_^c^	δ_H_^d^ (*J* in Hz)	δ_C_^e^	δ_H_^f^ (*J* in Hz)	δ_C_^g^
1	α: 1.10 m	α: 1.09 m	34.9	α: 1.90 m	23.0	α: 1.88 m	23.3
	β: 2.11 m	β: 2.09 m		β: 2.32 m		β: 2.32 m	
2	α: 1.80 m	α: 1.87 m	32.5	α: 1.69 m	29.7	α: 1.71 m	30.0
	β: 1.39 m	β: 1.41 m		β: 1.75 m		β: 1.75 m	
3	3.44 m	3.57 m	72.2	4.09 m	65.8	4.03 brs	66.1
4	2.28 m	α: 2.28 m	43.1	α: 2.20 m	36.7	α: 2.17 m	37.2
		β: 2.26 m		β: 2.40 m		β: 2.36 m	
5			137.2		125.8		124.7
6	5.62 d (5.2)	5.63 d (5.2)	126.7	3.83 brs	68.6	3.31 brs	78.1
7	α: 1.60 m	α: 1.65 m	31.7	α: 1.31 m	36.5	α: 1.07 m	30.8
	β: 2.32 m	β: 2.23 m		β: 1.82 m		β: 1.98 m	
8	2.13 m	2.08 m	30.0	1.50 m	23.0	1.48 m	33.4
9	1.02 m	1.02 m	52.1	1.49 m	46.5	1.45 m	46.4
10			41.1		135.4		135.6
11	1.58 m	α: 1.50 m	22.8	α: 1.84 m	25.1	α: 1.82 m	25.2
		β: 1.58 m		β: 1.24 m		β: 1.22 m	
12	α: 1.14 m	α: 1.12 m	42.7	α: 1.23 m	40.2	α: 1.21 m	40.2
	β: 2.00 m	β: 2.00 m		β: 2.03 m		β: 2.02 m	
13			43.3		43.1		43.1
14	0.82 m	0.83 m	63.3	1.16 m	54.8	1.13 m	54.9
15	4.14 td	4.18 td	70.5	α: 1.61 m	23.6	α: 1.62 m	23.6
	(5.6, 2.0)	(5.6, 2.0)		β: 1.16 m		β: 1.13 m	
16	α: 2.39 m	α: 2.43 m	42.1	α: 1.88 m	28.3	α: 1.88 m	28.3
	β: 1.34 m	β: 1.33 m		β: 1.30 m		β: 1.30 m	
17	1.10 m	1.09 m	57.6	1.17 m	56.1	1.16 m	56.2
18	0.97 s	0.97 s	15.1	0.71 s	12.2	0.70 s	12.2
19	4.02 d (12.0)	4.02 d (12.0)	65.6				
	4.53 d (12.0)	4.47 d (12.0)					
20	1.55 m	1.54 m	36.7	1.43 m	35.7	1.44 m	34.7
21	0.97 d (6.4)	0.96 d (6.4)	19.3	0.95 d (6.8)	18.6	0.95 d (6.5)	18.6
22	1.16 m	1.15 m	35.9	1.16 m	34.6	1.16 m	34.7
	1.58 m	1.54 m		1.55 m		1.56 m	
23	1.92 m	1.89 m	32.0	1.88 m	30.9	1.88 m	30.9
	2.11 m	2.08 m		2.10 m		2.11 m	
24			157.7		156.8		156.9
25	2.22 m	2.22 m	34.9	2.22 m	33.8	2.24 m	33.8
26	1.02 d (6.4)	1.03 d (6.8)	22.3	1.03 d (6.8)	22.0	1.03 d (7.0)	22.0
27	1.03 d (6.4)	1.03 d (6.8)	22.4	1.02 d (6.8)	21.9	1.03 d (7.0)	21.9
28	4.72 s	4.72 s	106.9	4.72 s	105.6	4.72 s	105.6
	4.66 s	4.66 s		4.66 s		4.66 s	
OAc	2.04 s	2.06 s	21.1				
			172.7				
OMe						3.34 s	57.0

^a^ Spectra were measured in CD_3_OD (400 MHz); ^b^ Spectra were measured in CDCl_3_ (400 MHz); ^c^ Spectra were measured in CD_3_OD (100 MHz); ^d^ Spectra were measured in CDCl_3_ (400 MHz); ^e^ Spectra were measured in CDCl_3_ (100 MHz); ^f^ Spectra were measured in CDCl_3_ (500 MHz); ^g^ Spectra were measured in CDCl_3_ (125 MHz).

**Figure 5:**
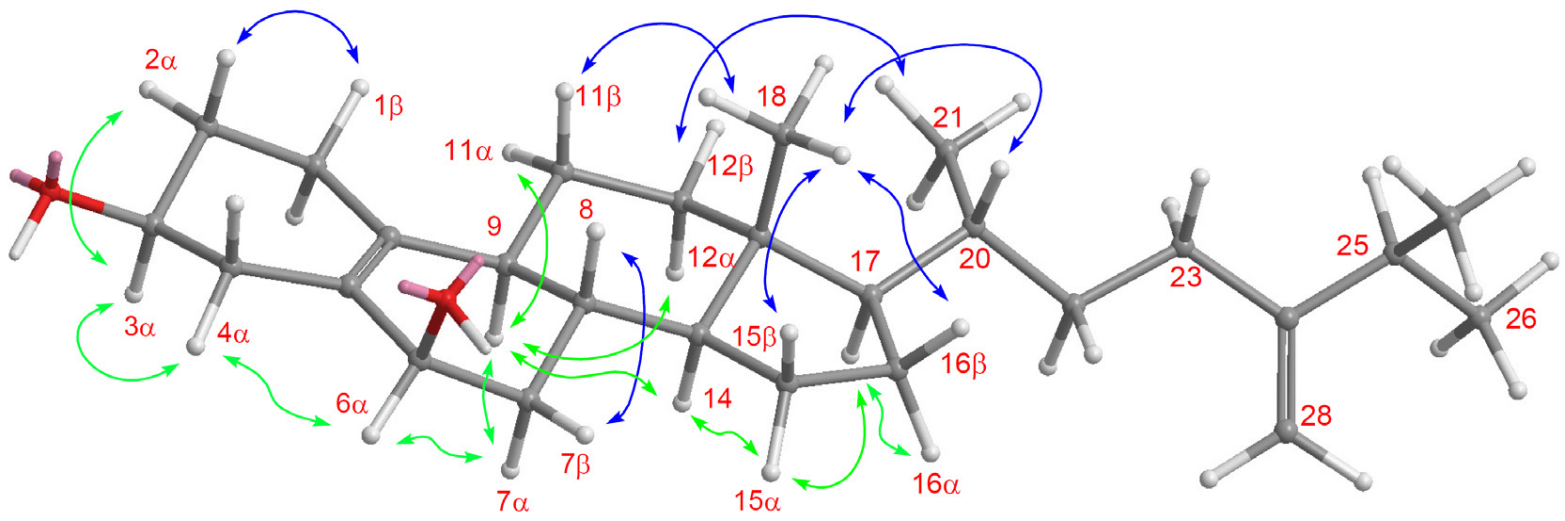
NOESY correlations of compound **2**.

Nebrosteroid R (**2**) was isolated as a white amorphous powder. HRESIMS of **2** exhibited a pseudo molecular ion peak at *m*/*z* 423.3241 [M + Na]^+^ (calcd for 423.3239) and established a molecular formula of C_27_H_44_O_2_, indicating six degrees of unsaturation. The ^13^C NMR (Table 1) displayed 27 carbon signals, which were identified by the assistance of the DEPT spectrum as four methyls, eleven methylenes, eight methines, and four quaternary carbons. The ^1^H NMR signal [δ_H_ 4.09 (m, 1H), 3.83 (brs, 1H)] (Table 1) and IR absorption at 3423 cm^−1^, together with the observation of two oxygen-bearing carbon resonances (δ_C_ 65.8 and 68.6) in the ^13^C NMR spectrum, revealed the presence of two hydroxyl groups. Furthermore, one tetrasubstituted double bond (δ_C_ 125.8 and 135.4), and one terminal double bond (δ_C_ 105.6 and 156.8) were assigned from ^13^C NMR and DEPT spectra of **2**. The above functionalities accounted for two of the six degrees of unsaturation, suggesting a tetracyclic skeleton for **2**. Interpretation of the ^1^H-^1^H COSY spectrum led to partial structures III and IV (Figure 3). The connectivities of these partial structures were further established by the HMBC correlations (Figure 3). Moreover, the COSY correlations from H_2_-1 to H-3 through H_2_-2 and from H-8 to H-6 through H_2_-7 led to the assignment of the secondary hydroxyl groups at C-3 and C-6. The location of the tetrasubstituted double bond at C-5/C-10 was clarified by analysis of the HMBC correlations from H_2_-6 to C-10, H_2_-2 to C-10, H-11 to C-10, and H-6 to C-5. The NOESY correlations (Figure 5) observed between H-3 and H-2α, H-3 and H-4α, H-4α and H-6α, H-6α and H-7α, H-7α and H-9, H-7β and H-8 indicated the β-orientations of the hydroxyl groups at C-3 and C-6. Moreover, the NOESY correlations observed between H-2β and H-1β, H-4α and H-6α, H-6α and H-7α, H-7α and H-9, H-7β and H-8, H-9 and H-14, H-11β and H-8, H-12β and Me-18, Me-18 and H-20, and Me-21 and H-12β in **2** confirmed the relative configurations for each ring junction and chiral center. Thus, the structure of **2** was established unambiguously.

Nebrosteroid R (**3**) was isolated as a white amorphous powder. HRESIMS of **3** exhibited a pseudo molecular ion peak at *m*/*z* 437.3398 [M + Na]^+^ (calcd for 437.3395) and established a molecular formula of C_28_H_46_O_2_, indicating six degrees of unsaturation. The ^13^C NMR (Table 1) displayed 28 carbon signals, which were identified with the assistance of the DEPT spectrum as five methyls, eleven methylenes, eight methines, and four quaternary carbons. The ^1^H NMR signal [δ_H_ 4.03 m (m, 1H)] (Table 1) and IR absorption at 3445 cm^−1^, together with the observation of one oxygen-bearing carbon resonance (δ_C_ 66.1) in the ^13^C NMR spectrum, revealed the presence of a secondary hydroxyl group. The ^1^H NMR signal [δ_H_ 3.31 (brs, 1H), 3.34 (s, 3H)] together with the observation of two oxygen-bearing carbon resonances (δ_C_ 57.0 and 78.1 in the ^13^C NMR spectrum, revealed the presence of a secondary methoxyl group. Furthermore, one tetrasubstituted double bond (δ_C_ 124.7 and 135.6), and one terminal double bond (δ_C_ 105.6 and 156.9) were assigned from ^13^C NMR and DEPT spectra of **3**. The above functionalities accounted for two of the six degrees of unsaturation, suggesting a tetracyclic skeleton for **3**. Interpretation of the ^1^H-^1^H COSY spectrum led to two similar partial structures as **2**. The connectivities of these partial structures were further established by HMBC correlations as **2**. Moreover, the COSY correlations from H_2_-1 to H-3 through H_2_-2 and from H-8 to H-6 through H_2_-7 as well as HMBC correlations from 6-OMe to H-6 led to the assignment of the secondary hydroxyl group at C-3 and the secondary methoxyl group at C-6. The location of the tetrasubstituted double bond at C-5/C-10 was clarified by analysis of the HMBC correlations from H_2_-6 to C-10, H_2_-2 to C-10, H-11 to C-10, and H-7 to C-5. The NOESY correlations observed between H-3 and H-2α, H-3 and H-4α, H-4α and H-6α, H-6α and H-7α, H-7α and H-9, H-7β and H-8 indicated the β-orientation of the hydroxyl group at C-3 and β-orientation of methoxyl group at C-6. Moreover, the NOESY correlations observed between H-2β and H-1β, H-4α and H-6α, H-6α and H-7α, H-7α and H-9, H-7β and H-8, H-9 and H-14, H-11β and H-12β, H-12β and Me-18, Me-18 and H-20, and Me-21 and H-12β in **3** confirmed the relative configurations for each ring junction and chiral center. Thus, the structure of **3** was established unambiguously. 

Nebrosteroids Q–S (**1**–**3**) were evaluated for cytotoxicity against P-388 (mouse lymphocytic leukemia), HT-29 (human colon adenocarcinoma), and A-549 (human lung epithelial carcinoma) tumor cells and the results are shown in Table 2. Nebrosteroids Q–S (**1**–**3**) exhibited cytotoxicity against P-388 cell line with ED_50_ of 1.1, 1.2, and 1.0 μg/mL, respectively. Nebrosteroids Q–S (**1**–**3**) were also examined for antiviral activity against human cytomegalovirus (HCMV) using a human embryonic lung (HEL) cell line. None was found to have anti-HCMV activity.

**Table 2:** Cytotoxicity and anti-human cytomegalovirus (HCMV) activities of **1**–**3**.

Compounds	ED_50_ (μg/mL)				Anti-HCMV (IC_50_; μg/mL)
	A549	HT-29	P-388	HEL	
**1**	6.1	8.0	1.1	>100	>100
**2**	11.4	20.9	1.2	>100	>100
**3**	8.7	15.3	1.0	>100	>100
Mithramycin	0.18	0.21	0.15	NT	NT
Ganciclovir	NT	NT	NT	NT	3.3

## 3. Experimental Section

### 3.1. General Experimental Procedures

Optical rotations were determined with a JASCO P1020 digital polarimeter. UV and IR spectra were obtained on JASCO V-650 and JASCO FT/IR-4100 spectrophotometers, respectively. NMR spectra were recorded on a Varian MR 400 NMR spectrometer at 400 MHz for ^1^H and 100 MHz for ^13^C or on a Varian Unity INOVA 500 FT-NMR spectrometer at 500 MHz for ^1^H and 125 MHz for ^13^C, respectively. ^1^H NMR chemical shifts are expressed in δ referring to the solvent peak δ_H_ 3.30 for CD_3_OD or δ_H_ 7.27 for CDCl_3_, and coupling constants are expressed in Hz. ^13^C NMR chemical shifts are expressed in δ referring to the solvent peak δ_H_ 49.0 for CD_3_OD or δ_C_ 77.0 for CDCl_3_. MS were recorded by a Bruker APEX II mass spectrometer. Silica gel 60 (Merck, Darmstadt, Germany, 230–400 mesh) and LiChroprep RP-18 (Merck, 40–63 μm) were used for column chromatography. Precoated silica gel plates (Merck, Kieselgel 60 F_254_, 0.25 mm) and precoated RP-18 F_254s_ plates (Merck) were used for thin-layer chromatography (TLC) analysis. High-performance liquid chromatography (HPLC) was carried out using a Hitachi L-7100 pump equipped with a Hitachi L-7400 UV detector at 220 nm together with a preparative reversed-phase column (Merck, Hibar LiChrospher RP-18e, 5 μm, 250 × 25 mm).

### 3.2. Biological Material

The soft coral *N. chabrolii* was collected by hand using scuba off the San-Hsian-Tai coast, Taitong County, Taiwan, in July 2009 at a depth of 6 m and stored in a freezer until extraction. The voucher specimen (SST-32) was identified by Prof. Chang-Feng Dai, National Taiwan University and deposited at the Department of Marine Biotechnology and Resources, National Sun Yat-sen University, Taiwan.

### 3.3. Extraction and Isolation

A specimen of soft coral *N. chabrolii* (2.2 kg) was minced and extracted with acetone (3 × 4 L) at room temperature. The combined acetone extracts were then partitioned between H_2_O and EtOAc. The resulting EtOAc extract (24.6 g) was subjected to gravity silica gel 60 column chromatography (Si 60 CC) using *n*-hexane and *n*-hexane/EtOAc of increasing polarity, to give 22 fractions. The fraction 12 (0.84 g), eluted with *n*-hexane/EtOAc (1:10), was further subjected to Si 60 CC (*n*-hexane/EtOAc, 10:1 to 100% EtOAc) to give six subfractions. A subfraction 12-2 (299 mg), was separated by a RP-18 flash column (MeOH/H_2_O, 50:50 to 100% MeOH) to give eight fractions. The subfraction 12-2-6, eluted with MeOH/H_2_O (90:10), was purified by RP-18 HPLC (MeOH/H_2_O, 95:5) to afford **1** (2.5 mg). The fraction 13 (0.69 g), eluted with EtOAc, was further subjected to Si 60 CC (*n*-hexane/EtOAc, 50:1 to 100% EtOAc) to give four subfractions. The subfraction 13-3 (299 mg), was separated by a RP-18 flash column (MeOH/H_2_O, 45:55 to 100% MeOH) to give three fractions. In turn, a subfraction 13-3-3, eluted with MeOH/H_2_O (90:10), was further purified by RP-18 HPLC (MeOH/H_2_O, 95:5) to afford **2** (3.0 mg) and **3** (1.0 mg).

Nebrosteroid Q (**1**): White amorphous powder; mp 176–177 °C [α]_D_^25^ −17.0 (*c* 0.1, CHCl_3_); IR (neat) ν_max_ 3375, 2926, 2853, 1737, 1640, 1593, 1363, 1240, 1039, 889 cm^−^^1^; ^1^H NMR (CD_3_OD, 400 MHz) and ^13^C NMR (CD_3_OD, 100 MHz) data in Table 1; HRESIMS *m*/*z* 495.3447 [M + Na]^+^ (calcd for C_30_H_48_O_4_Na, 495.3450).

Nebrosteroid R (**2**): White amorphous powder; mp 168–167 °C; [α]_D_^25^ +30.4 (*c* 0.1, CHCl_3_); IR (neat) ν_max_ 3423, 2926, 2853, 1640, 1596, 1458, 1378, 1042, 886 cm^−^^1^; ^1^H NMR (CDCl_3_, 400 MHz) and ^13^C NMR (CDCl_3_, 100 MHz) data in Table 1; HRESIMS *m*/*z* 423.3241 [M + Na]^+^ (calcd for C_27_H_44_O_2_Na, 423.3239).

Nebrosteroid S (**3**): White amorphous powder; mp 155–156 °C; [α]_D_^25^ +35.7 (*c* 0.1, CHCl_3_); IR (neat) ν_max_ 3445, 2922, 2851, 1640, 1456, 1380, 1081 cm^−^^1^; ^1^H NMR (CDCl_3_, 500 MHz) and ^13^C NMR (CDCl_3_, 125 MHz) data in Table 1; HRESIMS *m*/*z* 437.3398 [M + Na]^+^ (calcd for C_28_H_46_O_2_Na, 437.3395).

### 3.4. Cytotoxicity Assay

Cytotoxicity was determined on P-388 (mouse lymphocytic leukemia), HT-29 (human colon adenocarcinoma), and A-549 (human lung epithelial carcinoma) tumor cells using a modification of the MTT colorimetric method according to a previously described procedure [27,28]. The provision of the P-388 cell line was supported by J. M. Pezzuto, formerly of the Department of Medicinal Chemistry and Pharmacognosy, University of Illinois at Chicago. HT-29 and A-549 cell lines were purchased from the American Type Culture Collection. To measure the cytotoxic activities of tested compounds, five concentrations (50, 10, 2, 0.4, 0.08 μg/mL) with three replications were performed on each cell line. Mithramycin was used as a positive control.

### 3.5. Anti-HCMV Assay

To determine the effects of natural products upon HCMV cytopathic effect (CPE), confluent human embryonic lung (HEL) cells grown in 24-well plates were incubated for 1 h in the presence or absence of various concentrations of tested natural products with three replications. Ganciclovir was used as a positive control. Then, cells were infected with HCMV at an input of 1000 pfu (plaque forming units) per well of a 24-well dish. Antiviral activity was expressed as IC_50_ (50% inhibitory concentration), or compound concentration required to reduce virus induced CPE by 50% after seven days as compared with the untreated control. To monitor the cell growth upon treating with natural products, an MTT-colorimetric assay was employed [29].

## 4. Conclusion

This investigation of soft coral *N. chabrolii* collected at San-Hsian-Tai (Taitong County, Taiwan) has led to the isolation of a new cytotoxic 19-oxygenated steroid, nebrosteroid Q (**1**) and two new cytotoxic norergosterols, nebrosteroids R and S (**2** and **3**). Nebrosteroids Q–S (**1**–**3**) exhibited cytotoxicity against P-388 cell line with ED_50_ of 1.0, 1.2, and 1.0 μg/mL, respectively. However, previously isolated cholestene derivatives, nebrosteroids I–K [12] did not show cytotoxicity. In order to rule out the possibility of **3** being an isolation artifact, a solution of **2** was kept at room temperature for three days in the presence of Si-60 or RP-18 gel in MeOH. However, the formation of **3** was not observed.

## Supplementary Files

Supplementary File 1 Supplementary Information (PDF, 2760 KB)
