# Supplementary material for: New Steroids from the Soft Coral Nephthea chabrolii
Source: Mar Drugs. 2013 Feb 22;11(2):571–80. doi: 10.3390/md11020571 (PMC3640399; doi:10.3390/md11020571)

Supplementary Materials

Figure S1. ^{1}H NMR spectrum (400 MHz) of nebrosteroid Q (**1**) in CD_3OD. 2

Figure S2. ^{13}C NMR spectrum (100 MHz) of nebrosteroid Q (**1**) in CD_3OD. 3

Figure S3. COSY spectrum (400 MHz) of nebrosteroid Q (**1**) in CD_3OD. 4

Figure S4. HSQC spectrum (400 MHz) of nebrosteroid Q (**1**) in CD_3OD. 5

Figure S5. HMBC spectrum (400 MHz) of nebrosteroid Q (**1**) in CD_3OD. 6

Figure S6. NOESY spectrum (400 MHz) of nebrosteroid Q (**1**) in $CDCl_3$. 7

Figure S7. ^{1}H NMR spectrum (400 MHz) of nebrosteroid R (**2**) in $CDCl_3$. 8

Figure S8. ^{13}C NMR spectrum (100 MHz) of nebrosteroid R (**2**) in $CDCl_3$. 9

Figure S9. COSY spectrum (400 MHz) of nebrosteroid R (**2**) in $CDCl_3$. 10

Figure S10. HSQC spectrum (400 MHz) of nebrosteroid R (**2**) in $CDCl_3$. 11

Figure S11. HMBC spectrum (400 MHz) of nebrosteroid R (**2**) in $CDCl_3$. 12

Figure S12. NOESY spectrum (100 MHz) of nebrosteroid R (**2**) in $CDCl_3$. 13

Figure S13. ^{1}H NMR spectrum (500 MHz) of nebrosteroid S (**3**) in $CDCl_3$. 14

Figure S14. ^{13}C NMR spectrum (125 MHz) of nebrosteroid S (**3**) in $CDCl_3$. 15

Figure S15. COSY spectrum (500 MHz) of nebrosteroid S (**3**) in $CDCl_3$. 16

Figure S16. HSQC spectrum (500 MHz) of nebrosteroid S (**3**) in $CDCl_3$. 17

Figure S17. HMBC spectrum (500 MHz) of nebrosteroid S (**3**) in $CDCl_3$. 18

Figure S18. NOESY spectrum (500 MHz) of nebrosteroid S (**3**) in $CDCl_3$. 19

Figure S1. ^{1}H NMR spectrum (400 MHz) of nebrosteroid Q (**1**) in CD_3OD.

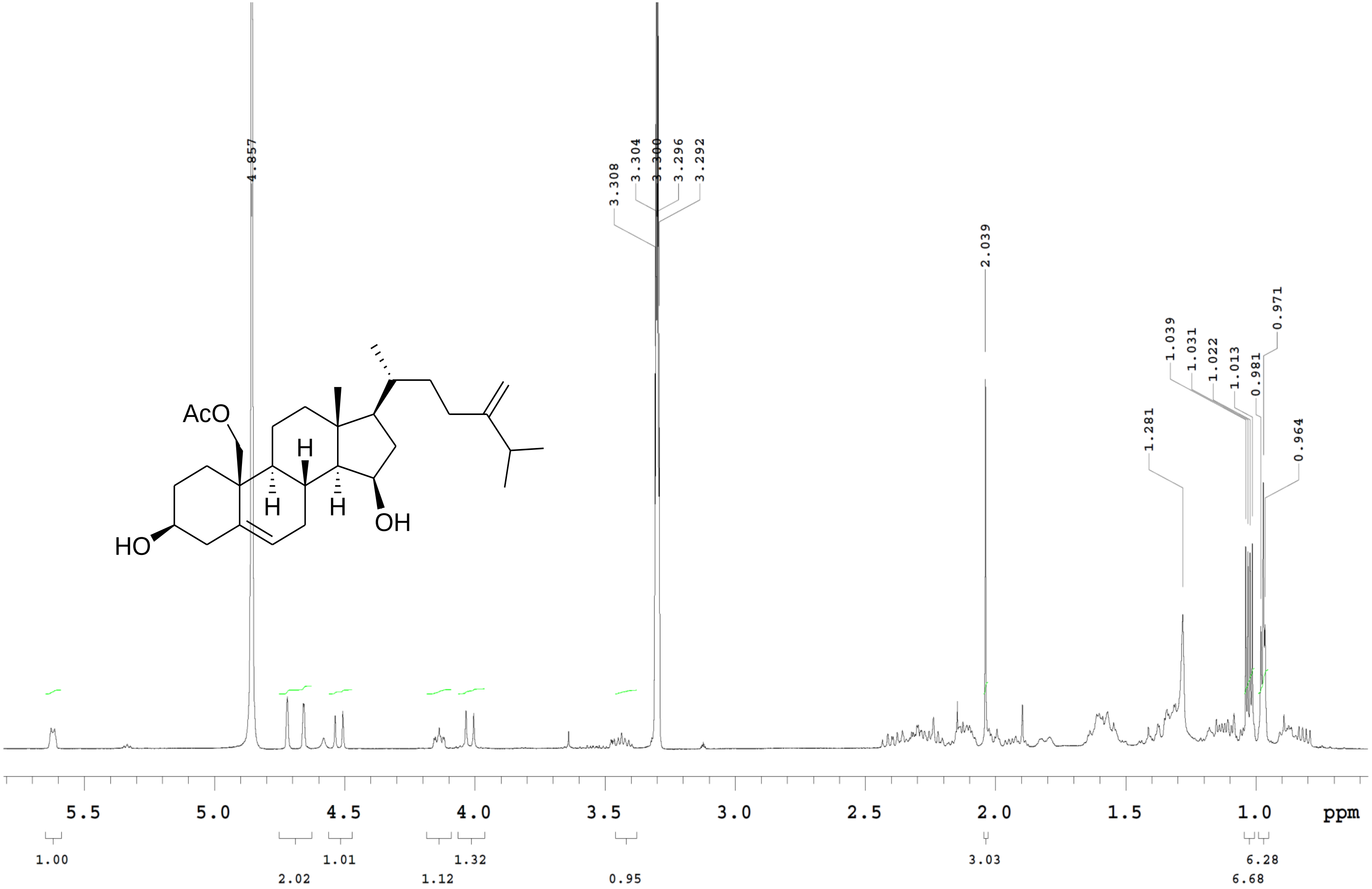

Figure S2. ^{13}C NMR spectrum (100 MHz) of nebrosteroid Q (**1**) in CD_3OD.

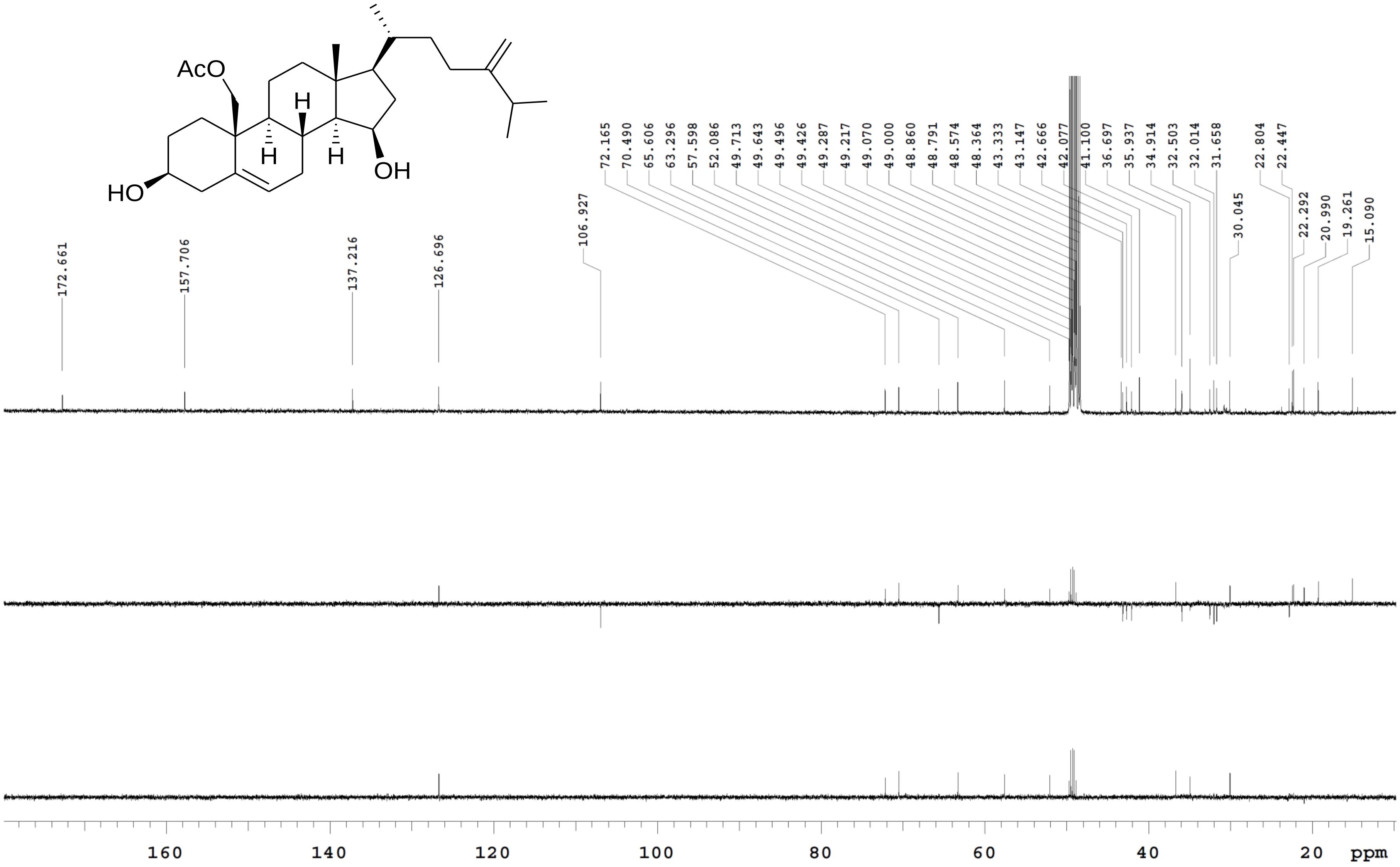

Figure S3. COSY spectrum (100 MHz) of nebrosteroid Q (**1**) in CD_3OD.

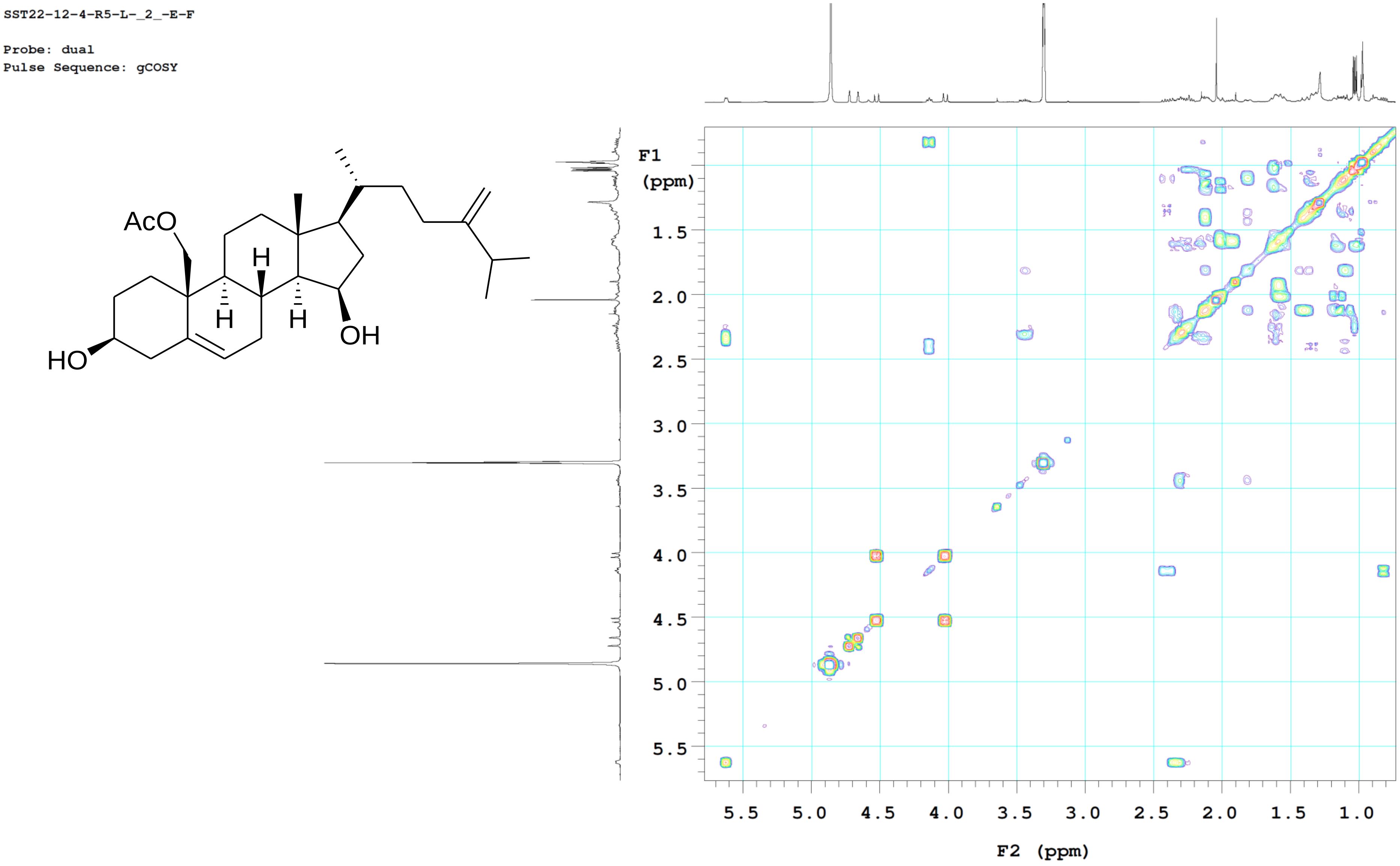

Figure S4. HSQC spectrum (100 MHz) of nebrosteroid Q (**1**) in CD_3OD.

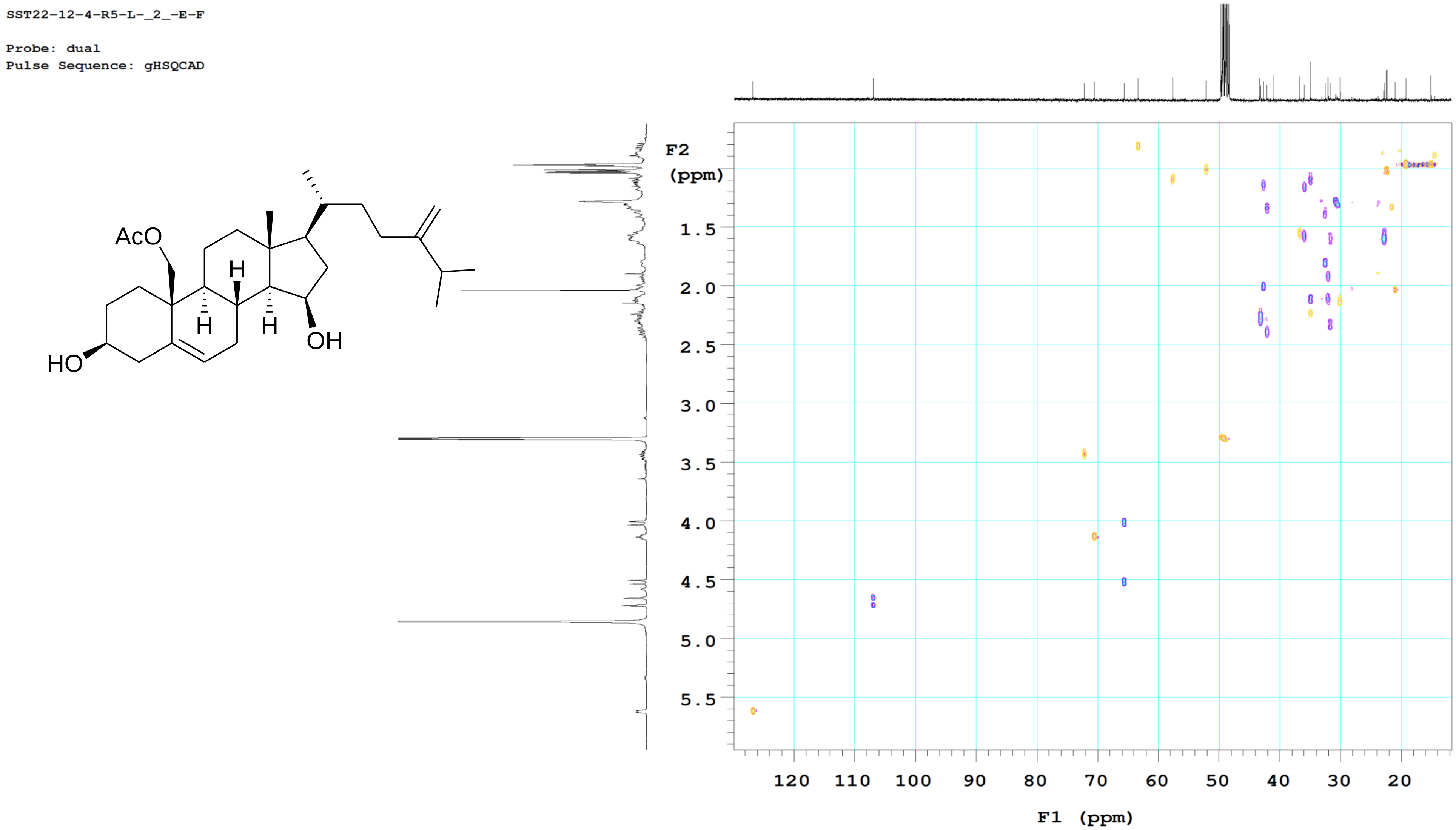

Figure S5. HMBC spectrum (100 MHz) of nebrosteroid Q (**1**) in CD_3OD.

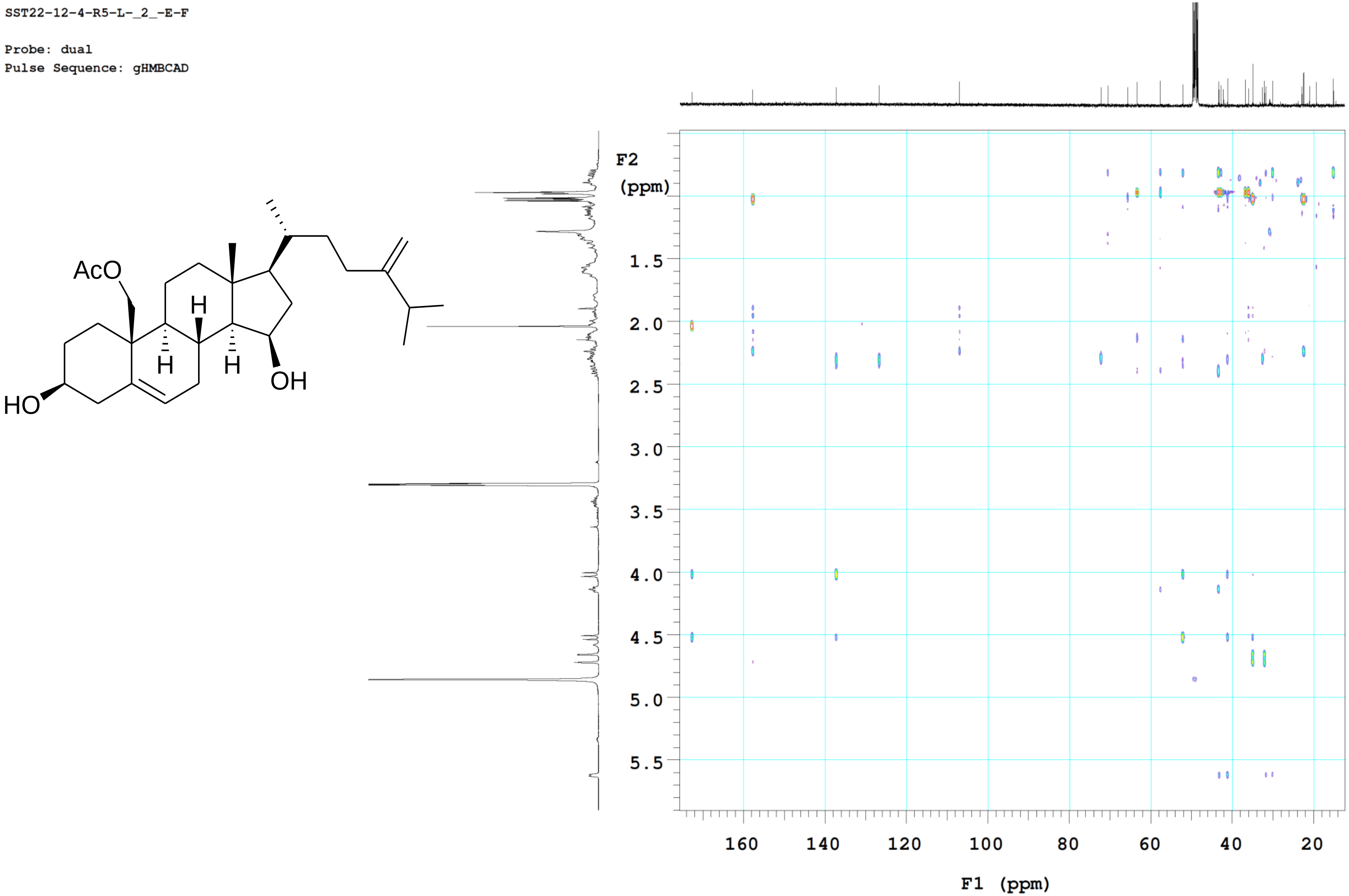

Figure S6. NOESY spectrum (100 MHz) of nebrosteroid Q (**1**) in $CDCl_3$.

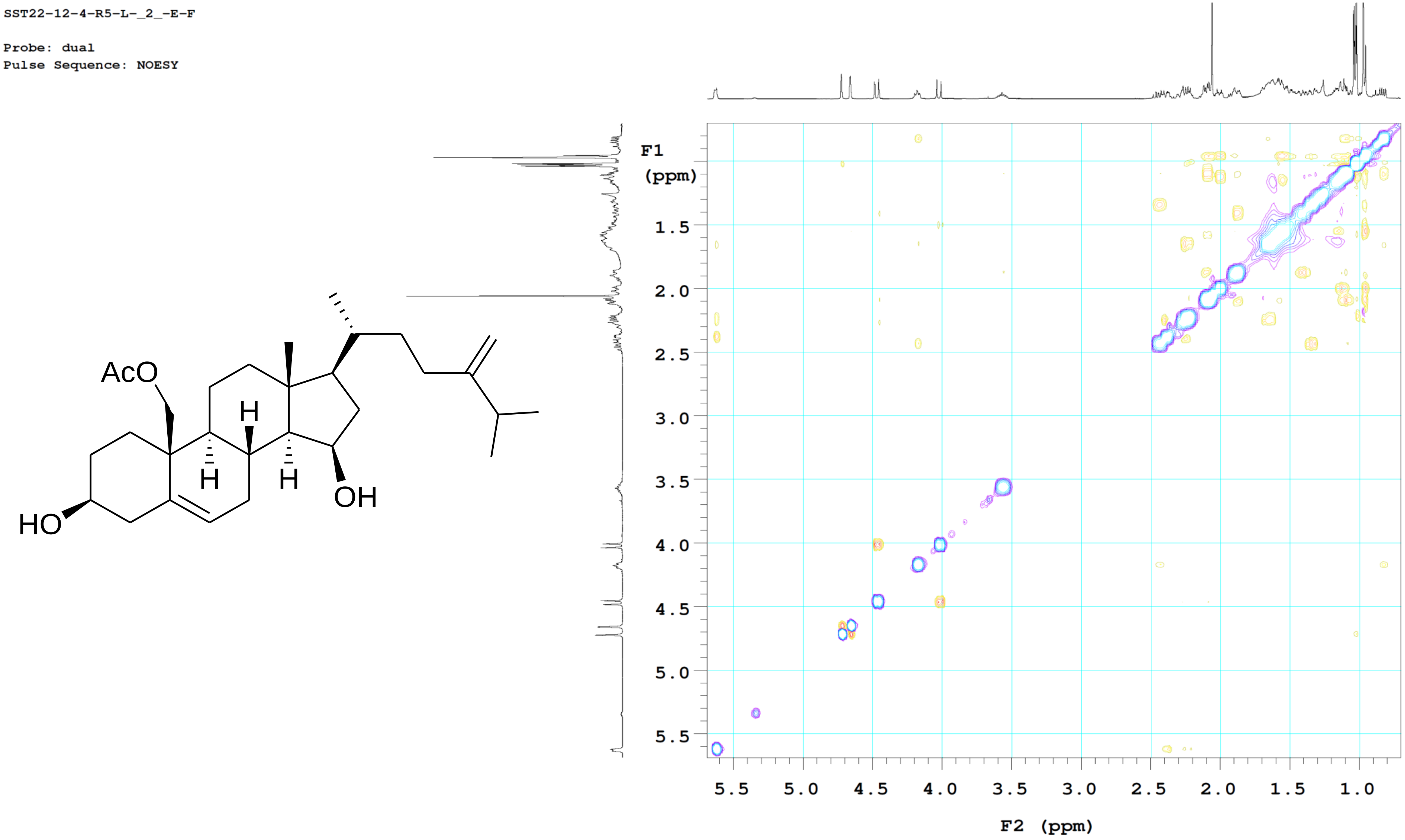

Figure S7. ^{1}H NMR spectrum (400 MHz) of nebrosteroid R (**2**) in $CDCl_3$.

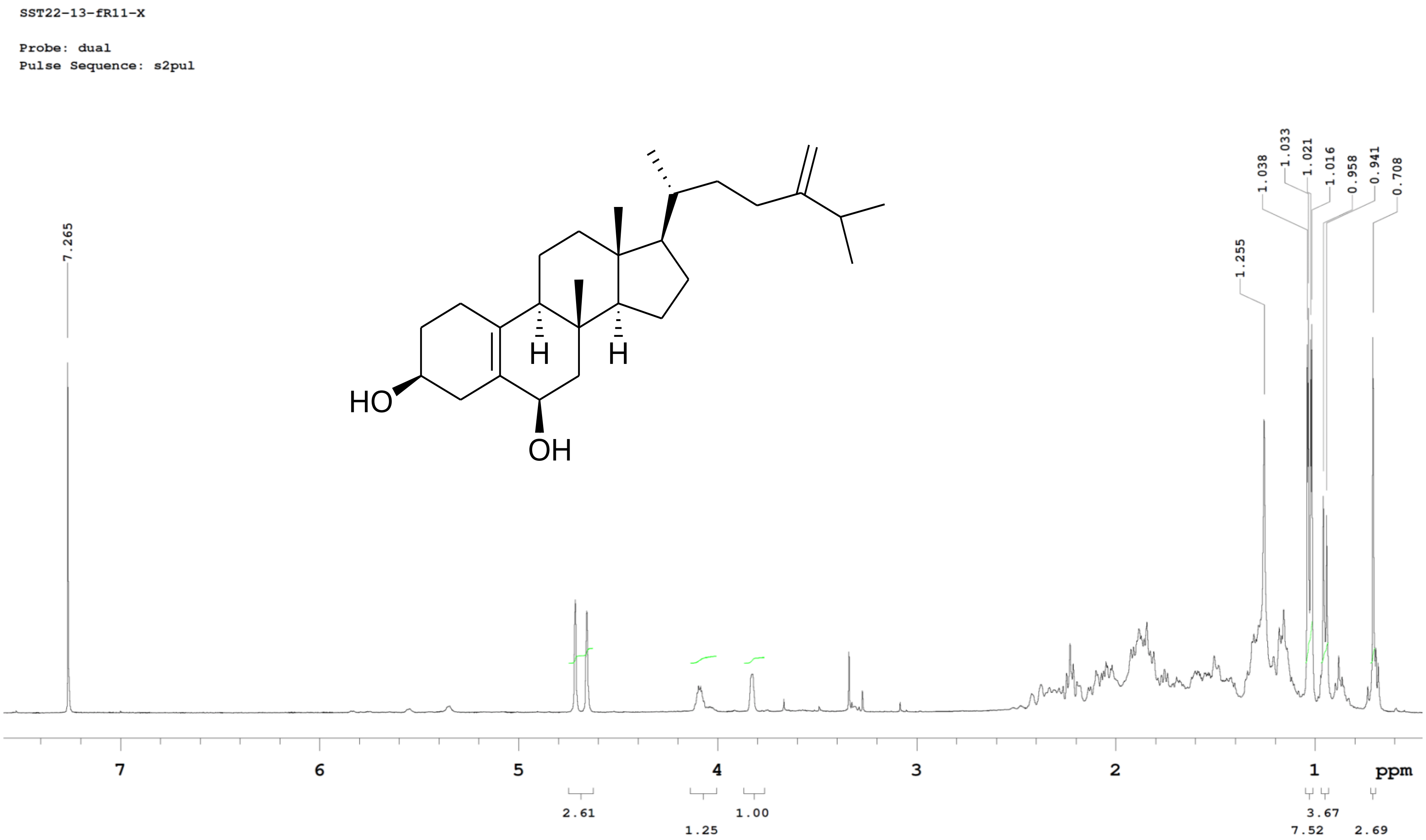

Figure S8. ^{13}C NMR spectrum (100 MHz) of nebrosteroid R (**2**) in $CDCl_3$.

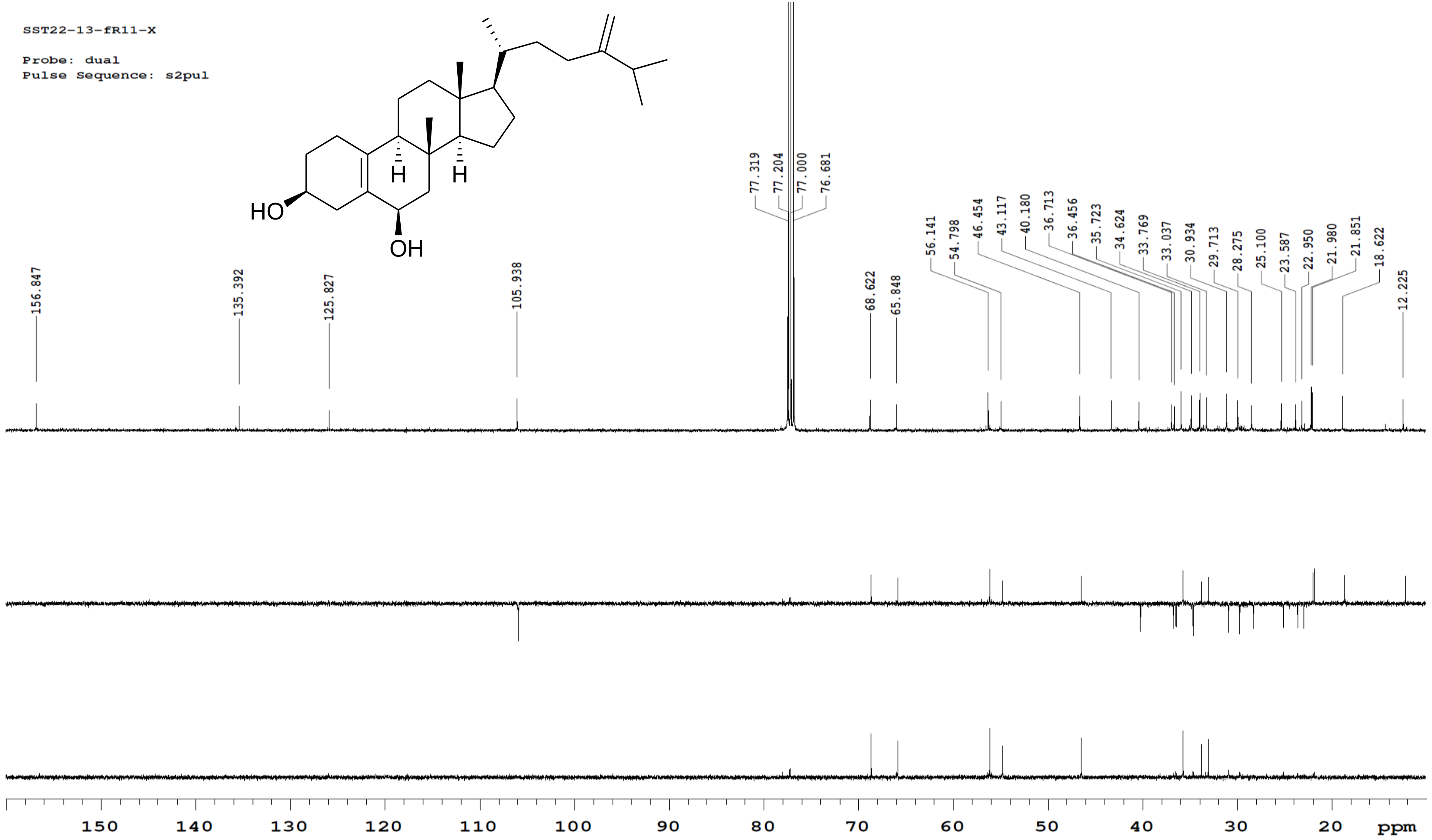

Figure S9. COSY spectrum (100 MHz) of nebrosteroid R (**2**) in $CDCl_3$.

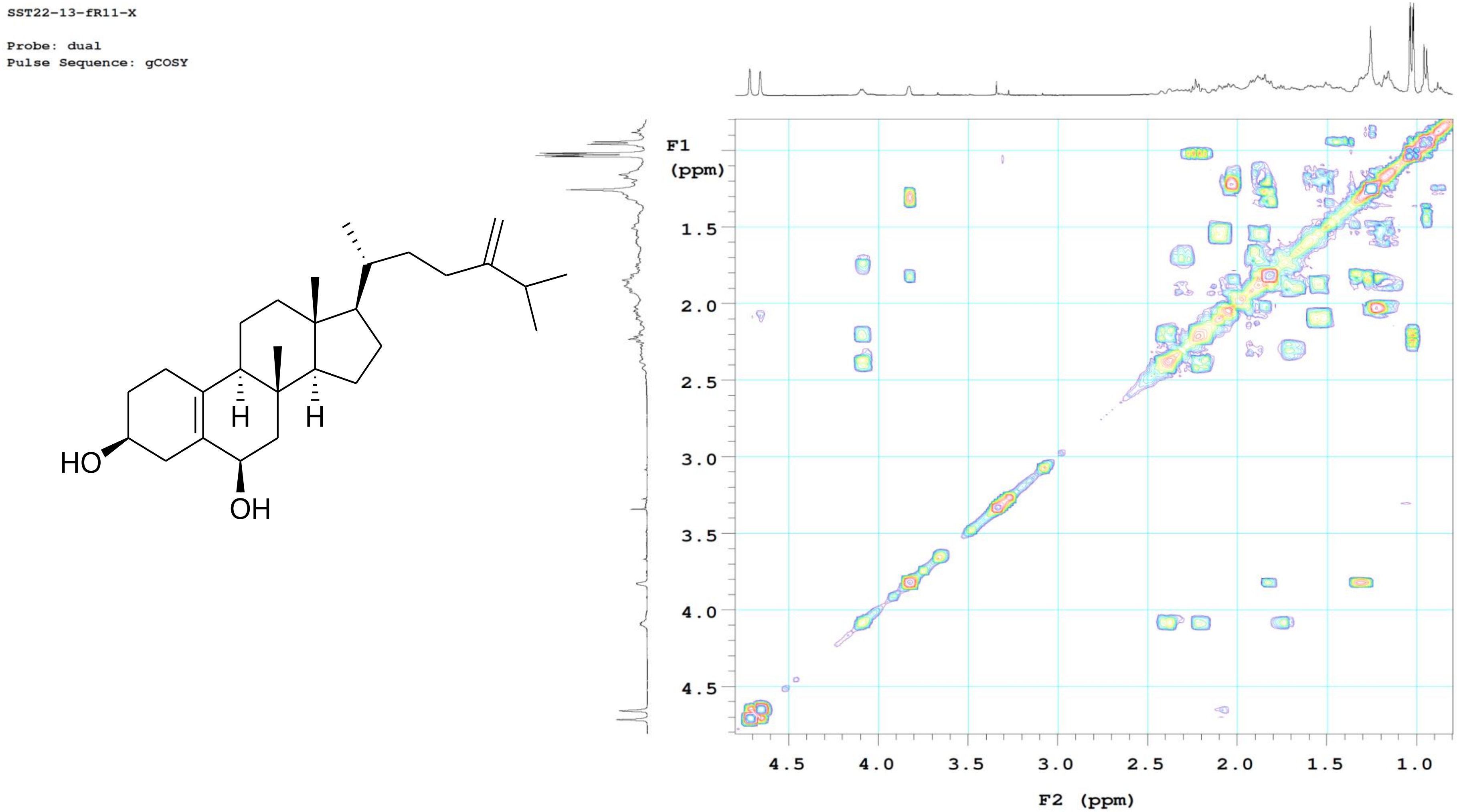

Figure S10. HSQC spectrum (100 MHz) of nebrosteroid R (**2**) in $CDCl_3$.

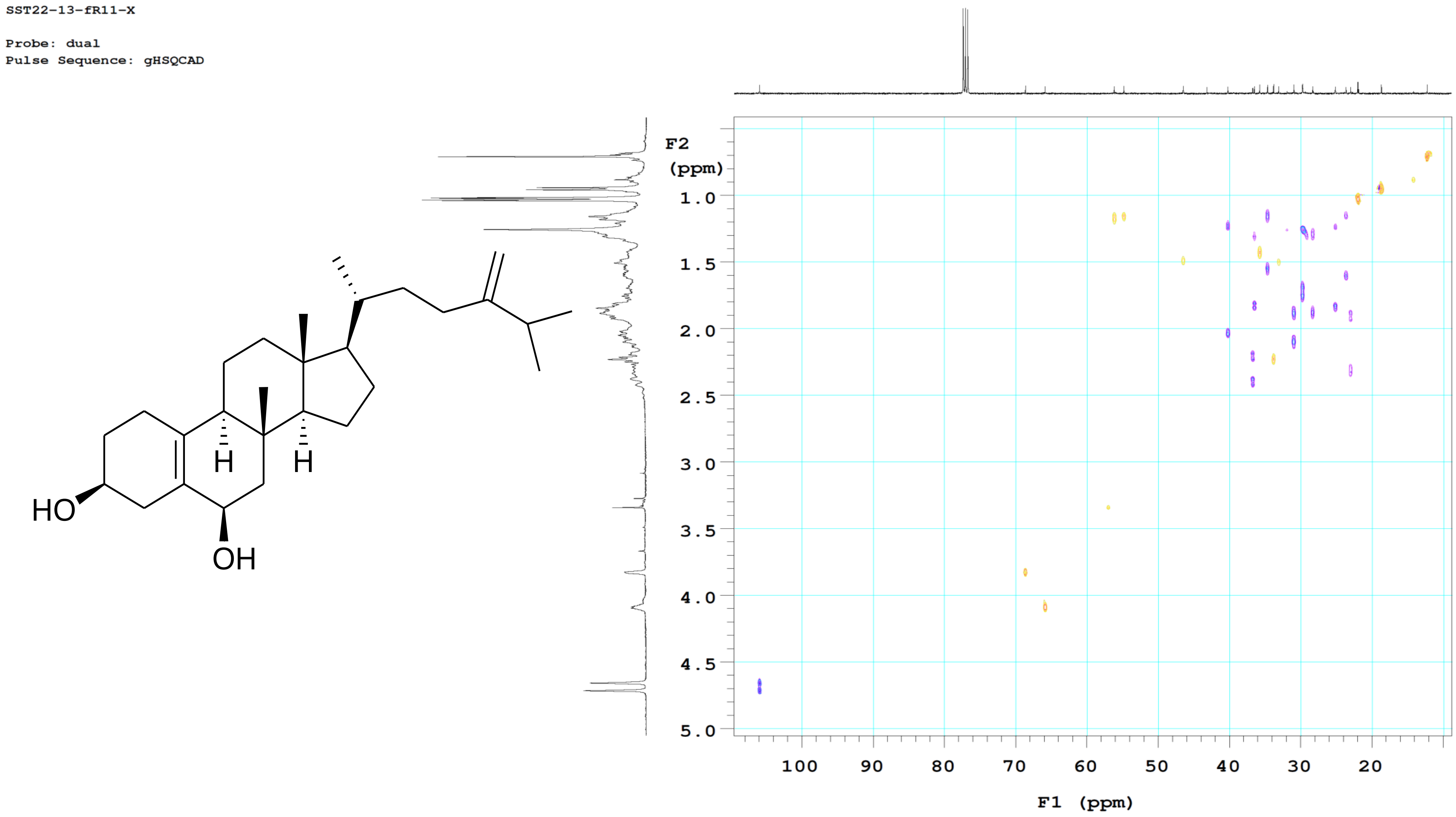

Figure S11. HMBC spectrum (100 MHz) of nebrosteroid R (**2**) in $CDCl_3$.

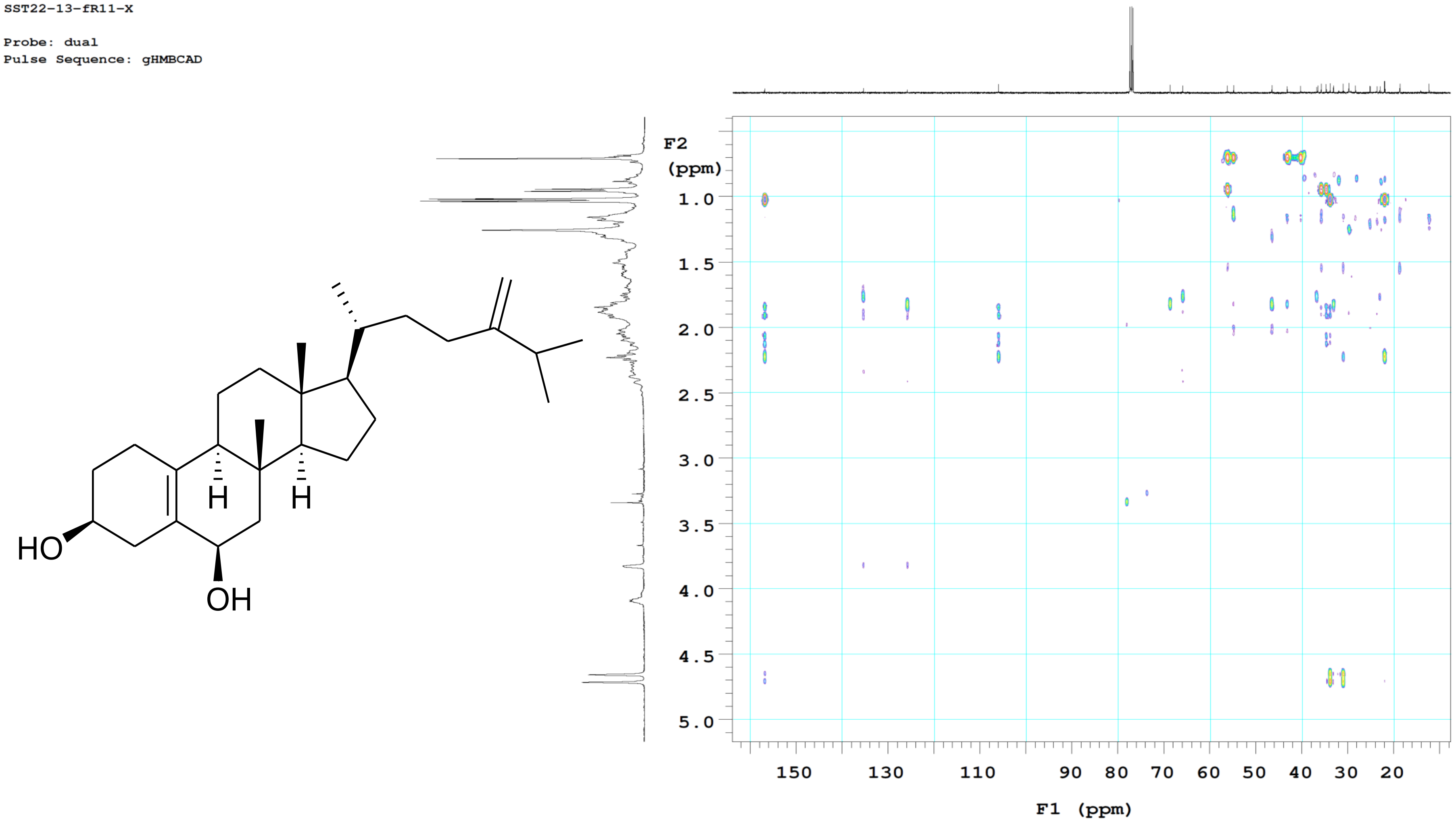

Figure S12. NOESY spectrum (100 MHz) of nebrosteroid R (**2**) in $CDCl_3$.

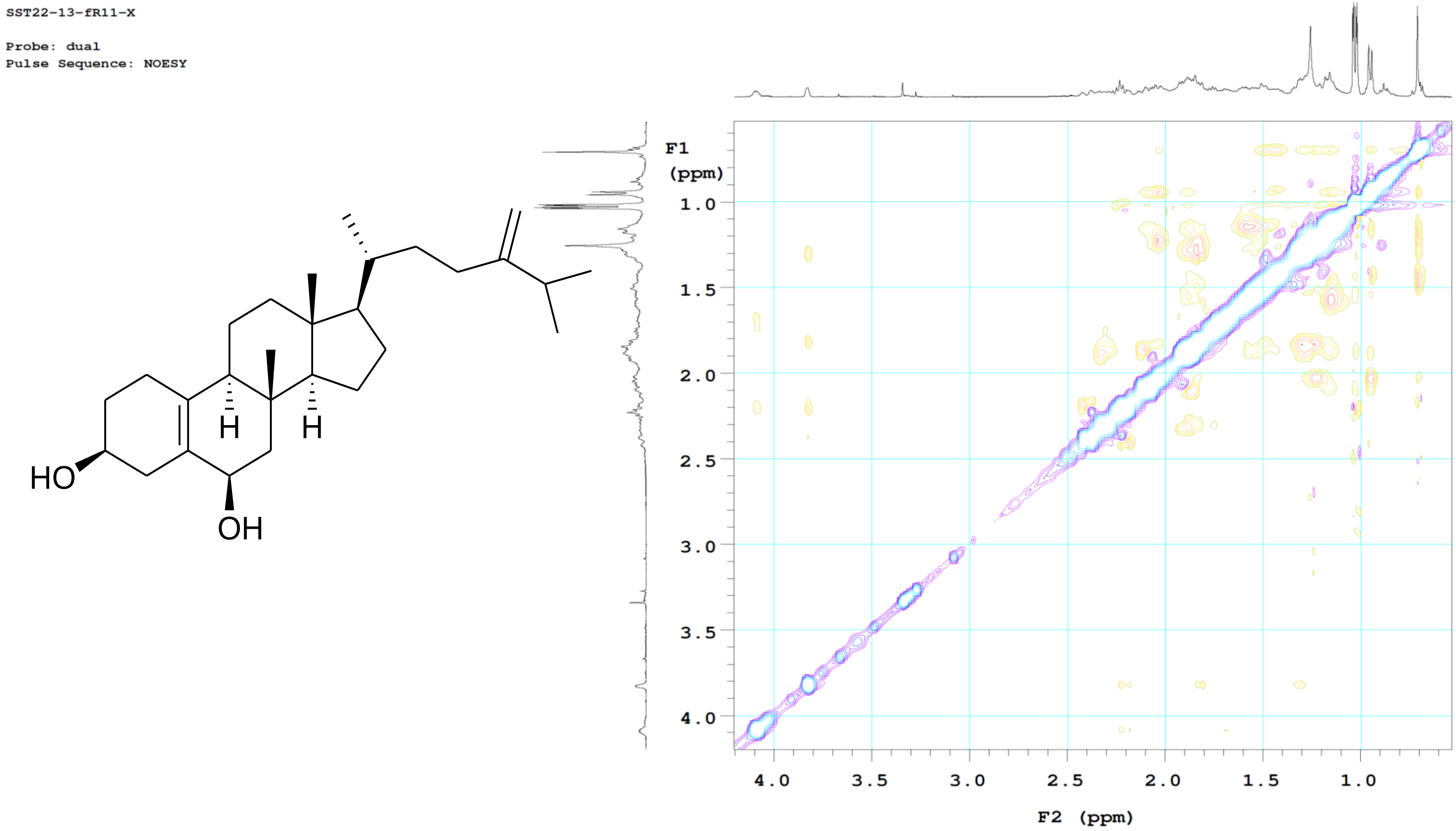

Figure S13. ^{1}H NMR spectrum (500 MHz) of nebrosteroid S (**3**) in $CDCl_3$.

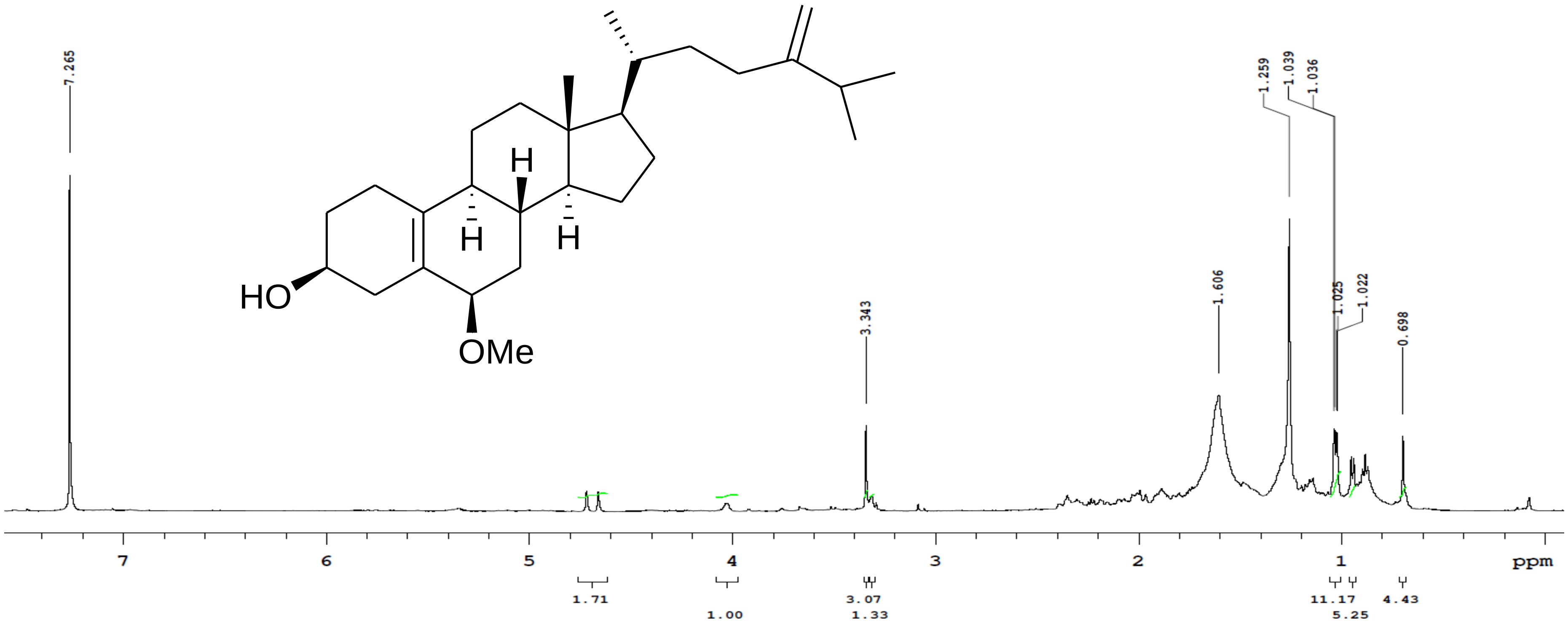

Figure S14. ^{13}C NMR spectrum (125 MHz) of nebrosteroid S (**3**) in $CDCl_3$.

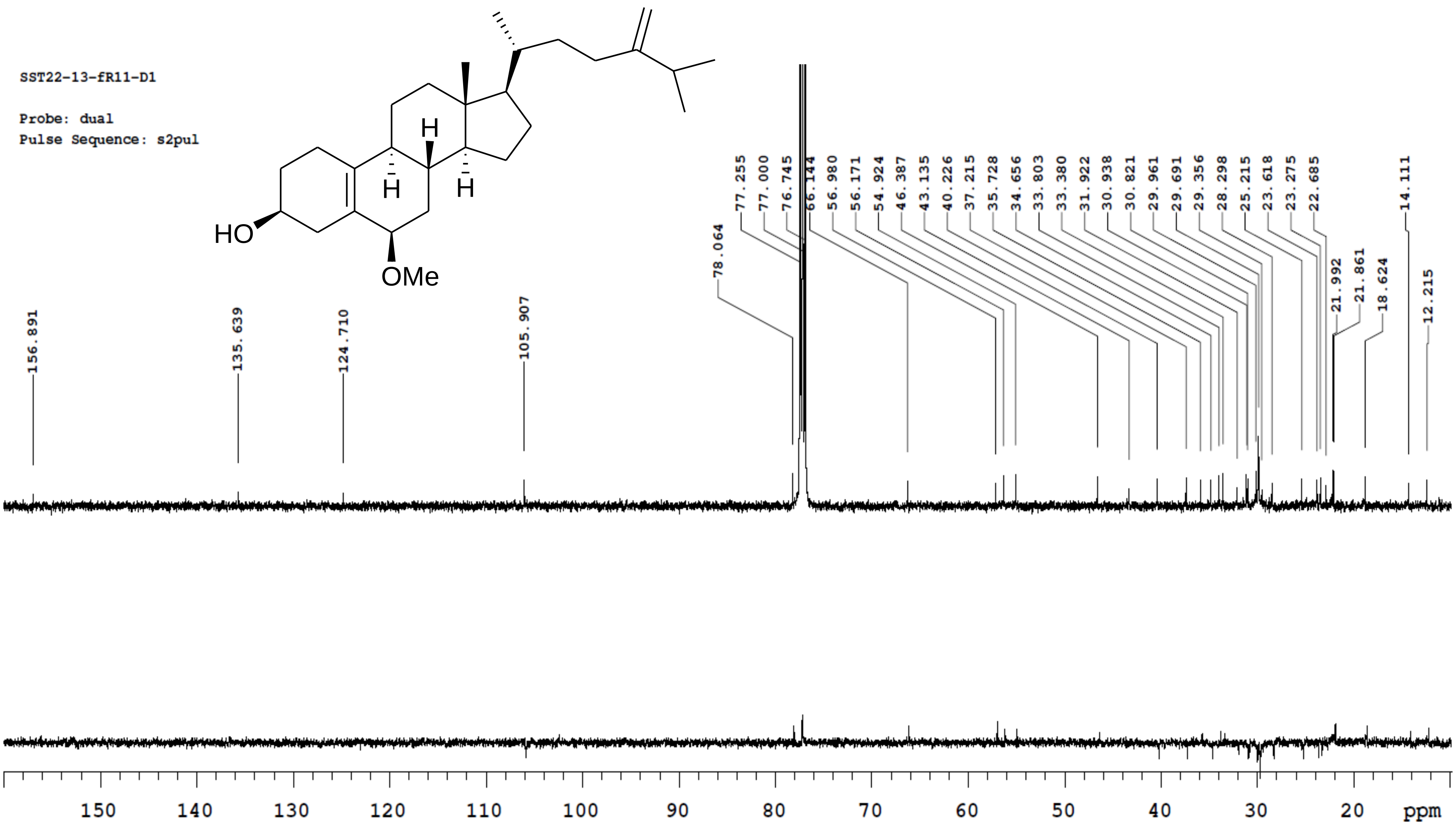

Figure S15. COSY spectrum (500 MHz) of nebrosteroid S (**3**) in $CDCl_3$.

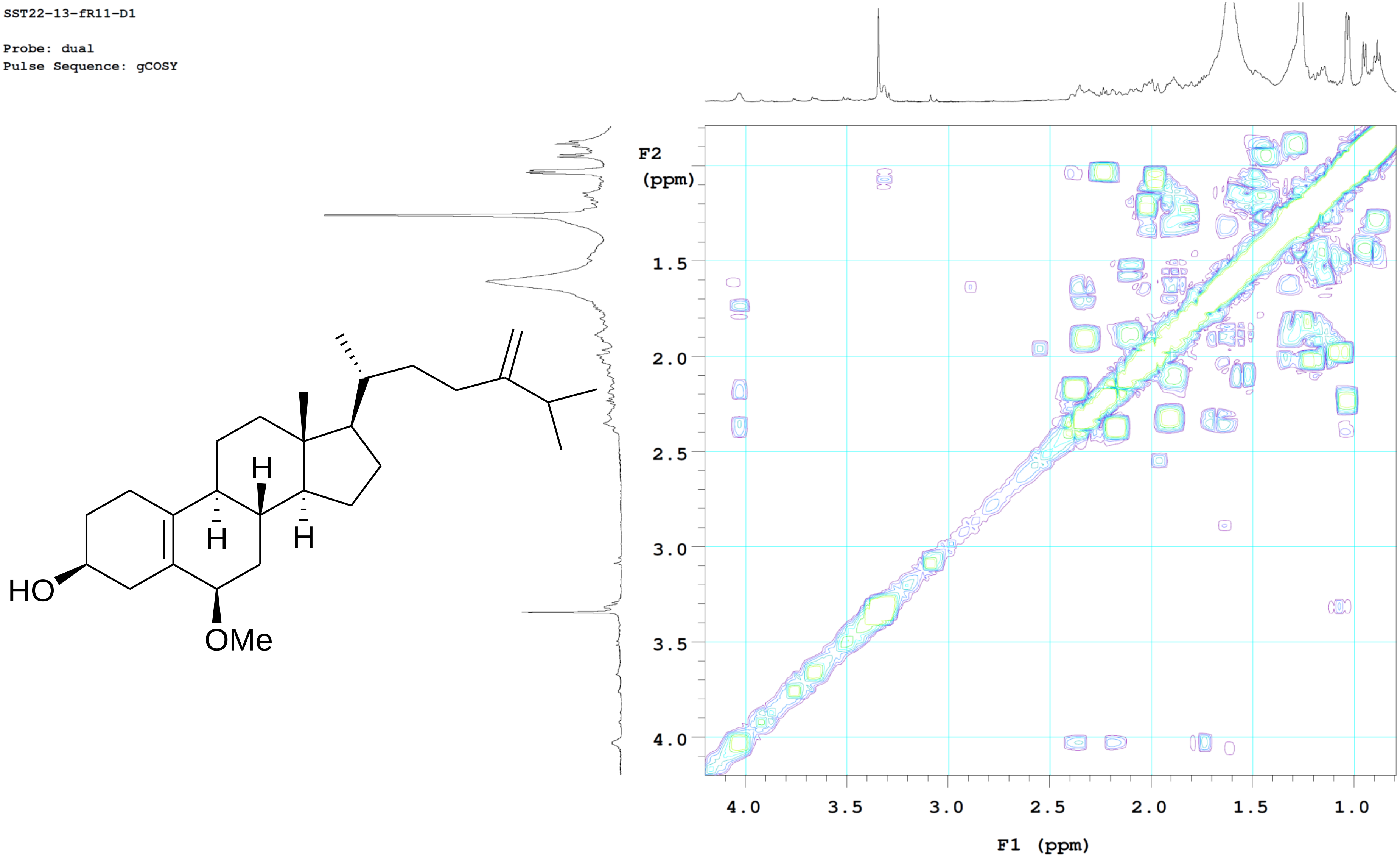

Figure S16. HSQC spectrum (500 MHz) of nebrosteroid S (**3**) in $CDCl_3$.

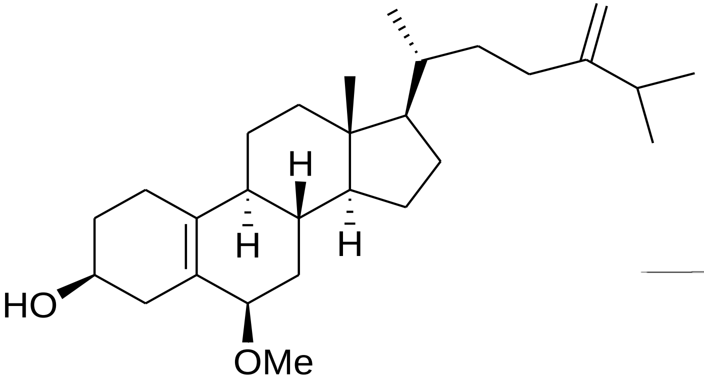

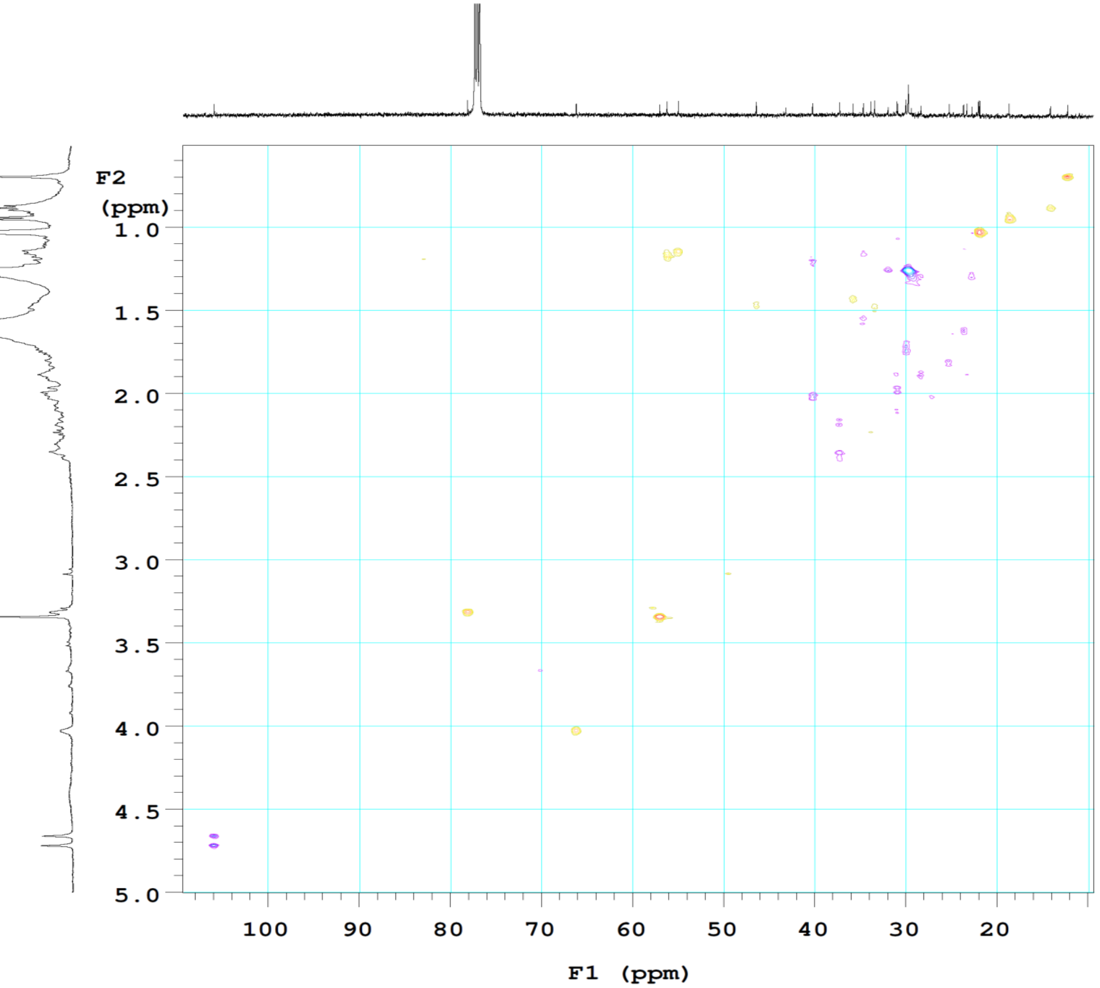

Figure S17. HMBC spectrum (500 MHz) of nebrosteroid S (**3**) in $CDCl_3$.

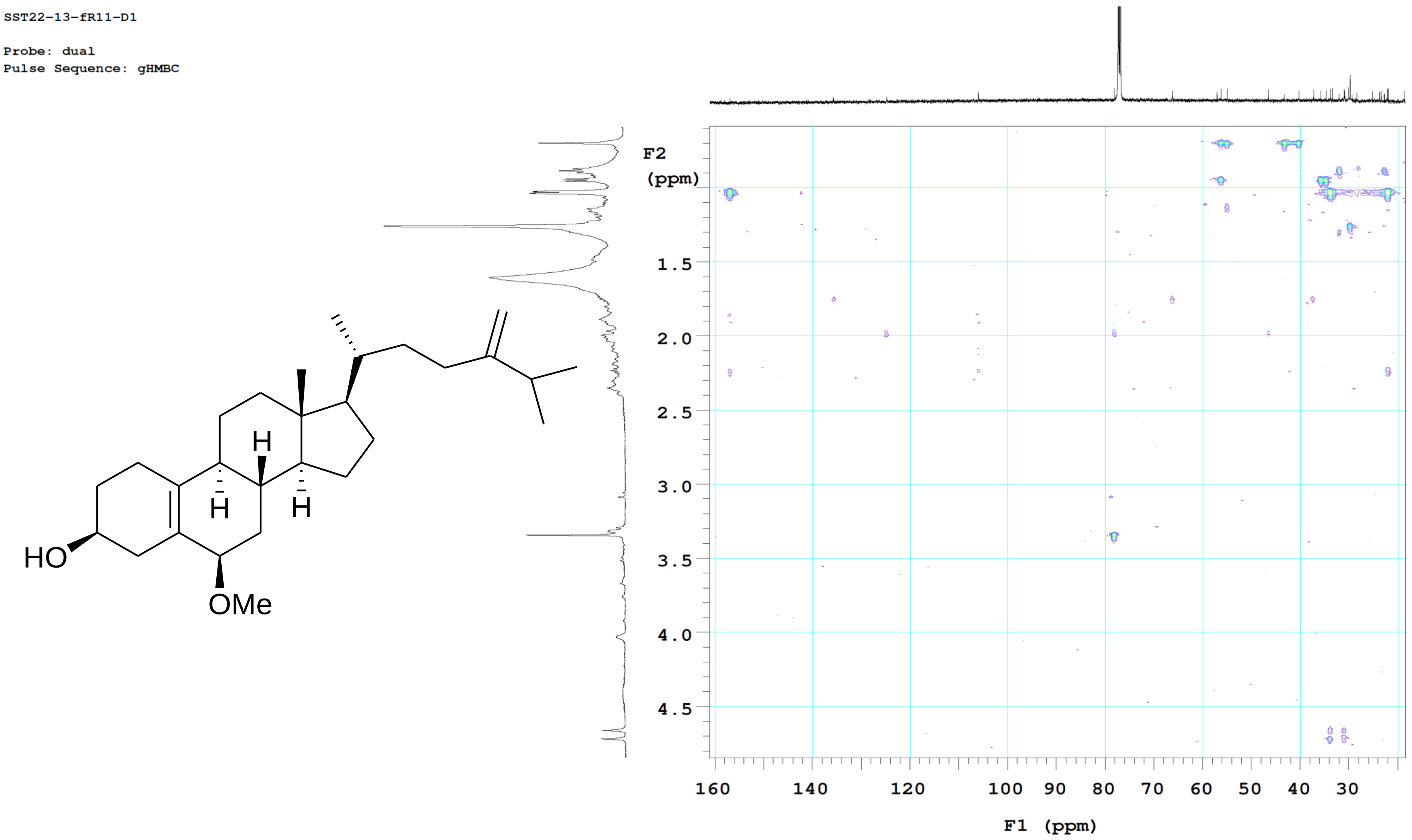

Figure S18. NOESY spectrum (500 MHz) of nebrosteroid S (**3**) in $CDCl_3$.

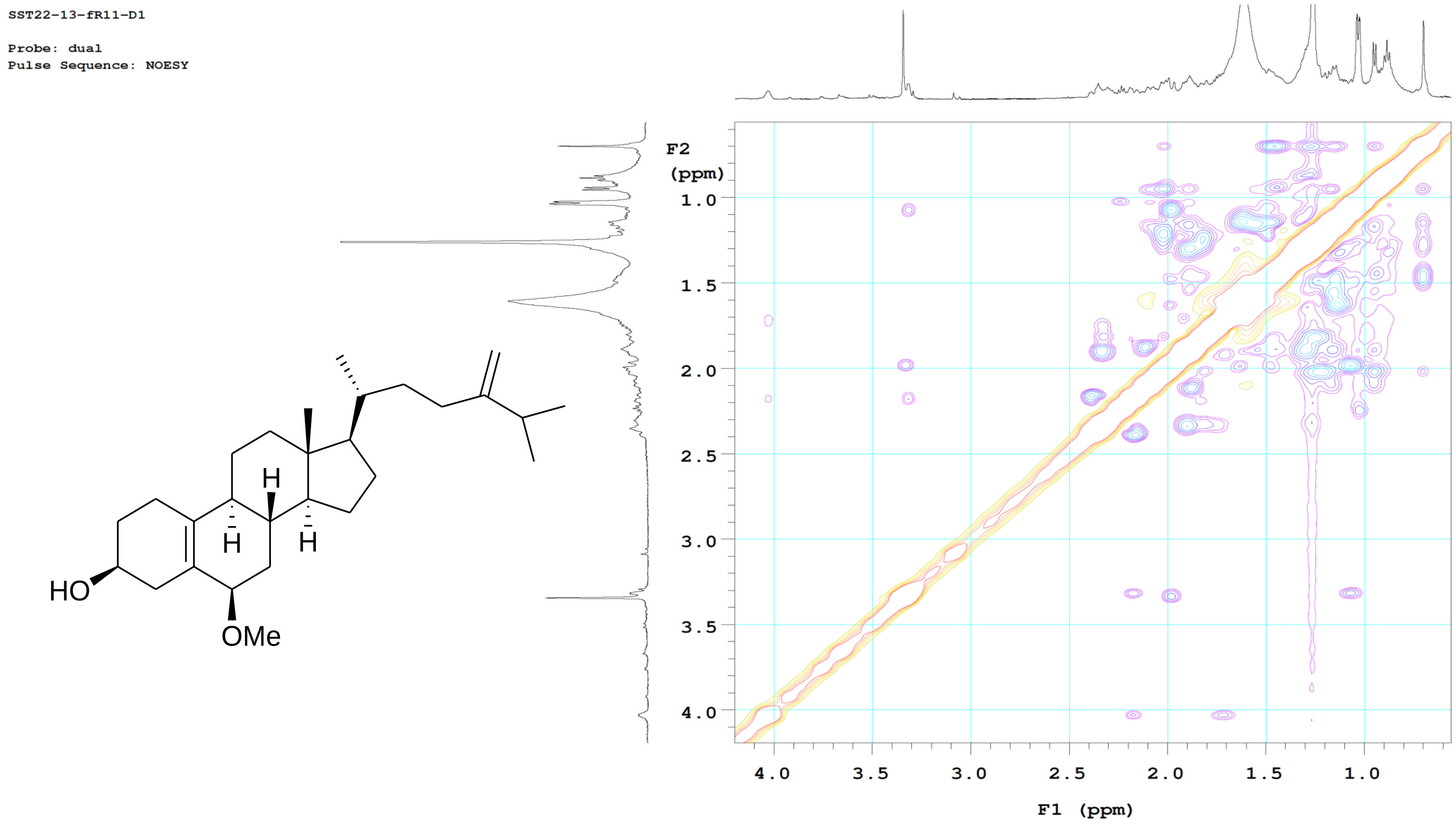

Supplement: Supplementary File 1 — Supplementary Information (PDF, 2760 KB) [file marinedrugs-11-00571-s001.pdf]
